# The pathogenesis of müller cell glial-mesenchymal transition in retinal fibrosis-related eye diseases

**DOI:** 10.3389/fcell.2026.1919772

**Published:** 2026-08-27

**Authors:** Jun Zhang, Jiaojiao Feng, Jike Song, Hongsheng Bi

**Affiliations:** 1 Ophthalmology & Optometry Medical School, Shandong University of Traditional Chinese Medicine, Jinan, China; 2 The First Clinical Medical College, Shandong University of Traditional Chinese Medicine, Jinan, China; 3 Shandong Provincial Key Laboratory of Integrated Traditional Chinese and Western Medicine for Prevention and Therapy of Ocular Diseases, Shandong Academy of Eye Disease Prevention and Therapy, Jinan, China; 4 Affiliated Eye Hospital of Shandong University of Traditional Chinese Medicine, Jinan, China

**Keywords:** cellular transdifferentiation, fibrogenic signaling, glial-mesenchymal transition, müller glial cells, retinal fibrosis

## Abstract

Retinal fibrosis represents an important pathological feature of several vision-threatening ocular diseases, including advanced diabetic retinopathy and neovascular age-related macular degeneration. Its progression may be associated with reactive changes in Müller glial cells and their acquisition of mesenchymal-like phenotypes. As the predominant glial population in the retina, Müller glial cells play crucial physiological roles in maintaining retinal homeostasis. However, under pathological conditions, they can become reactive and acquire mesenchymal- or myofibroblast-like features through a process referred to as glial–mesenchymal transition (GMT), potentially contributing to fibrotic remodeling. This review systematically examines the molecular mechanisms associated with Müller cell GMT, including the regulatory involvement of the TGF-β and Notch signaling pathways, inflammatory mediators, and oxidative stress. It discusses the potential contribution of GMT to retinal fibrotic progression. Furthermore, this article synthesizes the potential pathological and therapeutic relevance of GMT in related ocular diseases and discusses experimental intervention strategies targeting Müller cell phenotypic remodeling. Understanding the regulatory network of Müller cell GMT may inform the development of future therapeutic approaches to prevent or attenuate retinal fibrosis.

## Graphical Abstract

**Figure:**
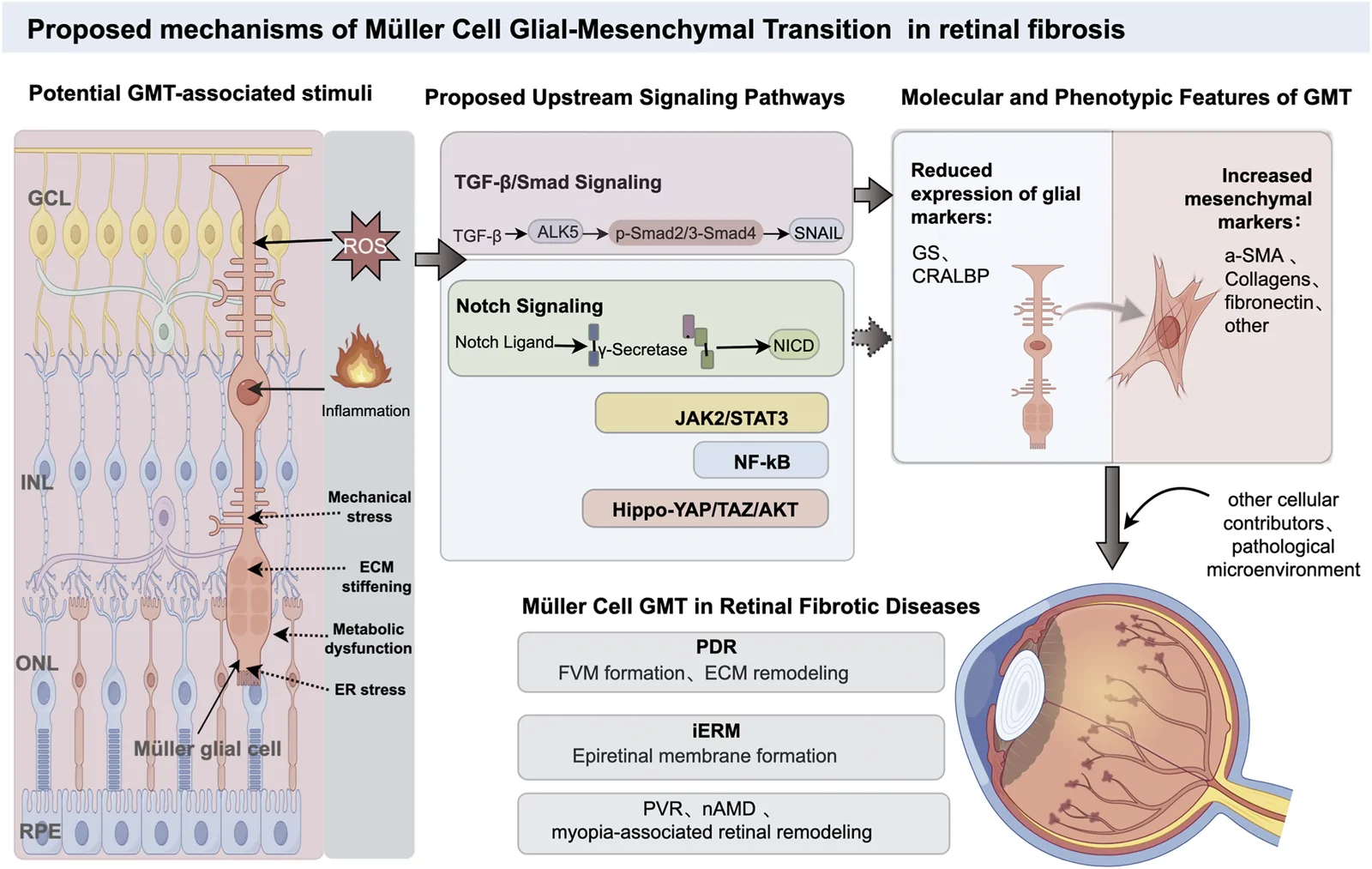
Proposed mechanisms of Müller cell GMT in retinal fibrosis. The figure summarizes the pathological stimuli and signaling pathways associated with GMT-like remodeling, the resulting molecular and functional phenotypes, and their potential contributions to retinal fibrotic membrane formation. Dashed arrows indicate proposed or incompletely validated relationships in Müller glial cells.

## 1 Introduction

Retinal fibrosis represents an important pathological outcome of several vision-threatening retinal diseases. Characterized by excessive deposition and remodeling of the extracellular matrix (ECM), formation of epiretinal or subretinal fibrotic membranes, and progressive distortion of retinal architecture, retinal fibrosis can cause irreversible visual impairment (Yi et al., 2022; Prieto-López et al., 2024). Over the past decade, research on retinal fibrosis has increasingly progressed from descriptive pathology toward mechanistic investigation of the cellular origins and signaling networks involved in fibrotic remodeling. Evidence from animal models, human single-cell transcriptomic studies, and *in vitro* systems indicates that retinal fibrosis is not merely a passive scar-forming process but an active, multicellular remodeling process involving phenotypic and functional changes in retinal, vascular, immune, and stromal cell populations.

The mechanisms underlying retinal fibrosis involve interactions among inflammatory stimuli, mechanical stress, oxidative injury, and cytokine-driven signaling pathways. Among the various retinal cell types implicated, Müller glia may participate in the development or amplification of fibrotic remodeling (Prieto-López et al., 2024). As the principal macroglial population spanning the entire retina, Müller glia are responsive to microenvironmental perturbations and can adopt heterogeneous reactive states following injury. Emerging evidence suggests that reactive Müller glia may acquire mesenchymal-like molecular and functional features, a process referred to as glial–mesenchymal transition (GMT) (Kanda et al., 2019; Krishna Chandran et al., 2021). GMT-like remodeling is associated with altered expression of canonical glial markers, increased expression of mesenchymal- and ECM-associated molecules, and changes in cellular migration and contractility (Kanda et al., 2019; Krishna Chandran et al., 2021). These changes may contribute to the formation or remodeling of fibrotic membranes, although the causal and relative contribution of Müller cell GMT to retinal fibrosis *in vivo* remains to be established. Elucidating the molecular mechanisms regulating Müller cell GMT may therefore improve our understanding of retinal fibrotic remodeling and inform the identification of potential therapeutic targets.

## 2 Retinal müller glial cells

Müller glial cells constitute the principal macroglial population of the mammalian retina. Their specialized radial morphology enables extensive physical interactions with multiple retinal cell types across different retinal layers, including photoreceptors, bipolar cells, retinal ganglion cells (RGCs), endothelial cells, pericytes, and other glial populations, whereas their metabolic adaptations support continuous biochemical exchange with these cells (Medina-Arellano et al., 2024; Bringmann et al., 2006). Müller glia maintain retinal homeostasis by regulating extracellular ion and water balance, clearing neurotransmitters such as glutamate, and providing trophic and antioxidant support to retinal neurons (Pereiro et al., 2022; Carpi-Santos et al., 2022). They also contribute to retinal energy metabolism by storing and mobilizing glycogen and supporting neuronal activity through metabolic substrate exchange, including lactate metabolism (Carpi-Santos et al., 2022). Moreover, their interactions with endothelial cells and pericytes contribute to neurovascular coupling and the maintenance of inner blood–retinal barrier integrity (Medina-Arellano et al., 2024; Miyata et al., 2021).

Müller glia exhibit marked interspecies differences in their responses to retinal injury. In regeneration-competent vertebrates, particularly zebrafish, Müller glia can reprogram into retinal progenitor-like cells and subsequently generate multiple retinal neuronal subtypes, thereby supporting retinal regeneration (Yin et al., 2025; Conedera et al., 2021). In contrast, mammalian Müller glia possess limited spontaneous neurogenic capacity and respond to injury predominantly by adopting reactive states (Conedera et al., 2021; da Silva et al., 2023; Sardar Pasha et al., 2017). This divergence has been associated with differences in neurogenic transcriptional programs, including Ascl1 expression, as well as YAP-mediated regulation of cell-cycle progression and regenerative responses (Ueki et al., 2015; Hamon et al., 2019). In the mammalian retina, reactive gliosis in Müller glia is a common but heterogeneous response to injury or pathological stress. It involves cellular hypertrophy, altered expression of intermediate filaments such as GFAP and vimentin, metabolic remodeling, and changes in the production of inflammatory, trophic, and angiogenic mediators (da Silva et al., 2023; Miao et al., 2023; Liu et al., 2022). Hyperglycemia, TRPV4 activation, oxidative stress, and endoplasmic reticulum stress have been reported to promote selected features of Müller glial reactivity in experimental models, whereas PRDX4 and thioredoxin-1 may attenuate some of these changes by reducing endoplasmic reticulum or oxidative stress (Huang et al., 2025; Yu et al., 2023). Depending on the disease context and the intensity and duration of injury, reactive Müller glia may support neuronal survival and tissue homeostasis or contribute to persistent inflammation, impaired metabolic support, vascular dysfunction, and structural remodeling. Reactive gliosis should therefore be regarded as a heterogeneous and context-dependent injury response rather than an inherently protective or detrimental process.

Retinal astrocytes constitute another important macroglial population and share several homeostatic and injury-responsive properties with Müller glia, including neuronal support, neurovascular regulation, and reactive gliosis (Yoo et al., 2021; de Hoz et al., 2016). However, the two populations occupy distinct anatomical and functional niches. Retinal astrocytes are located predominantly in the nerve fiber and ganglion cell layers, where they commonly express GFAP under physiological conditions and interact closely with RGC axons and the superficial vasculature. In contrast, Müller glia extend radially across the full thickness of the neural retina and typically show marked GFAP upregulation following injury (Bringmann et al., 2006; de Hoz et al., 2016). Their radial organization and extensive interactions with neuronal, vascular, and ECM compartments may enable Müller glia to integrate pathological signals arising from different retinal layers. These anatomical and functional characteristics provide an important biological context for understanding the potential involvement of Müller glia in mesenchymal-like remodeling under pathological conditions. However, they do not by themselves establish that Müller glia are more susceptible than retinal astrocytes to GMT or that Müller cell GMT is sufficient to drive retinal fibrosis.

## 3 Glial-mesenchymal transition

Glial–mesenchymal transition (GMT) has been proposed as an EMT-like form of phenotypic remodeling in which glial cells acquire mesenchymal- or myofibroblast-like molecular and functional features. Epithelial–mesenchymal transition (EMT) is a biological process in which epithelial cells lose epithelial polarity and cell–cell adhesion while acquiring mesenchymal properties, including increased motility and ECM-remodeling capacity. The GMT concept was initially developed mainly in the context of glioma and was subsequently applied to non-neoplastic glial responses during tissue injury, remodeling, and fibrosis (Lamouille et al., 2014; Mahabir et al., 2014). However, GMT is not identical to classical EMT because glial cells are not epithelial cells and therefore do not undergo canonical loss of epithelial polarity or epithelial cell–cell junctions. In the context of Müller glia, GMT refers more specifically to the acquisition of a mesenchymal-like transcriptional and functional phenotype, including altered expression of canonical glial markers, increased expression of α-SMA, fibronectin, collagens, and other mesenchymal-associated molecules, as well as enhanced migration, contractility, and ECM synthesis or remodeling (Kanda et al., 2019; Krishna Chandran et al., 2021; da Silva et al., 2023; Hart et al., 2024).

Importantly, Müller cell GMT should be distinguished from reactive gliosis. Reactive gliosis is a broad and heterogeneous stress-response program. The molecular and functional states of reactive Müller glia vary according to the nature and duration of injury, disease stage, anatomical location, and local microenvironment (Lamas and Martinez-Colin, 2022; Hippert et al., 2015). GMT, in contrast, implies coordinated acquisition of mesenchymal- or myofibroblast-like molecular and functional properties (Kanda et al., 2019; Krishna Chandran et al., 2021; da Silva et al., 2023; Hart et al., 2024). GMT-like changes may arise within a subset of reactive Müller glia exposed to persistent profibrotic stimulation, and intermediate states may retain glial markers while acquiring selected mesenchymal features (Kanda et al., 2019; Wu et al., 2020). Reactive gliosis and Müller cell GMT are not necessarily mutually exclusive or completely separate processes: they share several injury-associated triggers and may coexist but differ in the extent of mesenchymal-associated feature acquisition and in the molecular and functional evidence required for their identification (Table 1). Moreover, studies of reactive astrocytes have shown that glial responses encompass heterogeneous inflammatory, neurotoxic-like, anti-inflammatory, and reparative-like states depending on disease context, timing, and the local microenvironment (Yoo et al., 2021). Although these astrocyte-derived classifications should not be directly extrapolated to Müller glia, transcriptomic studies have similarly identified Müller glial subpopulations with distinct inflammatory, metabolic, angiogenic, homeostatic, or tissue-remodeling profiles under physiological and pathological conditions (Liu et al., 2022; Menon et al., 2019; Wang et al., 2022; Zhang et al., 2023). However, no specific Müller glial subpopulation has yet been shown to preferentially undergo GMT. Findings from certain fibrotic models suggest that macrophages exhibiting M2-like tissue-remodeling features may be more permissive to macrophage-to-myofibroblast transition (MMT) (Ban et al., 2024), raising the hypothesis that Müller glial states enriched in reparative or tissue-remodeling programs might likewise show increased responsiveness to profibrotic stimuli. This analogy remains speculative and should not be considered evidence of a corresponding Müller glial state. Whether pre-existing or disease-induced heterogeneity influences Müller glial responsiveness to TGF-β, inflammatory mediators, oxidative stress, or other profibrotic stimuli remains a hypothesis requiring single-cell and spatial analyses combined with lineage-tracing and functional validation.

**TABLE 1:** Comparison between reactive gliosis and GMT in Müller glia.

Characteristic	Reactive gliosis	GMT	References
Process	A broad and heterogeneous stress-response program involving morphological, molecular, metabolic, and functional remodeling	Proposed coordinated acquisition of mesenchymal- or myofibroblast-like molecular and functional features	(Kanda et al., 2019; Bringmann et al., 2009; Lewis and Fisher, 2003)
Major triggers	Retinal injury, neuronal degeneration, ischemia, inflammation, oxidative stress, metabolic disturbance, or mechanical stress	Sustained profibrotic stimulation, particularly TGF-β signaling, potentially modified by inflammatory, oxidative, metabolic, and mechanical cues	(Kanda et al., 2019; Bringmann et al., 2006; Wu et al., 2020; Bringmann et al., 2009)
Main molecular features	Altered GFAP and vimentin expression, with context-dependent changes in nestin, GS, CRALBP, GLAST, inflammatory mediators, and stress-response genes	Increased expression of α-SMA, SM22, fibronectin, collagens, SNAIL, and other mesenchymal-associated molecules, accompanied by reduced or altered expression of selected glial markers	(Kanda et al., 2019; Wu et al., 2020; Bringmann et al., 2009; Lewis and Fisher, 2003)
Functional outcome	Context-dependent changes in neuronal support, metabolic adaptation, inflammatory signaling, tissue remodeling, and retinal homeostasis, with consequences varying according to the nature, severity, and duration of injury	Increased migration, contractility, and ECM production or remodeling, as reported mainly in cultured Müller glia; these changes may contribute to fibrocellular or fibrovascular membrane formation	(Kanda et al., 2019; Krishna Chandran et al., 2021; Bringmann et al., 2006; Wu et al., 2020; Bringmann et al., 2009)
Reversibility	Dynamic and potentially reversible in some contexts following resolution of injury, although severe or prolonged reactive states may persist	Not established in Müller glia; the stability and reversibility of partial or persistent GMT-like states require longitudinal lineage-tracing and functional studies	(Krishna Chandran et al., 2021; Bringmann et al., 2009)
Relationship to fibrosis	Not inherently fibrotic; may precede or accompany fibrosis but does not itself establish mesenchymal transition	Associated with profibrotic molecular and functional features, although GMT-like markers alone do not demonstrate a causal contribution to retinal fibrosis	(Kanda et al., 2019; Krishna Chandran et al., 2021; Wu et al., 2020)
Representative evidence	Müller glial hypertrophy and altered GFAP or vimentin expression following retinal degeneration, detachment, ischemia, diabetes, or light-induced injury	Detection or colocalization of glial and mesenchymal-associated markers in PDR fibrovascular membranes and iERMs, together with TGF-β-induced molecular, migratory, contractile, and ECM-related changes in cultured Müller glia	(Kanda et al., 2019; Krishna Chandran et al., 2021; Wu et al., 2020; Lewis and Fisher, 2003)

Abbreviations: α-SMA, α-smooth muscle actin; CRALBP, cellular retinaldehyde-binding protein; ECM, extracellular matrix; GFAP, glial fibrillary acidic protein; GLAST, glutamate–aspartate transporter; GMT, glial–mesenchymal transition; GS, glutamine synthetase; iERM, idiopathic epiretinal membrane; PDR, proliferative diabetic retinopathy; SM22, smooth muscle protein 22; TGF-β, transforming growth factor-β.

Increasing evidence indicates that Müller glia can acquire GMT-like molecular and phenotypic features under chronic or severe pathological stress, including ischemic injury and proliferative retinopathies. These changes have been associated with retinal fibrosis and tissue remodeling, although direct evidence establishing Müller cell GMT as a causal driver of fibrosis *in vivo* remains limited. Gillem et al. (1998) reported that cultured rabbit retinal Müller glia underwent progressive phenotypic changes during serial passaging. Early-passage cells expressed vimentin, GFAP, GS, and β-amyloid precursor protein, whereas later-passage cells exhibited increased α-SMA expression accompanied by loss of GS, GFAP, and vimentin. These findings demonstrate substantial Müller glial plasticity *in vitro*; however, the extent to which serial passaging reproduces phenotypic changes occurring during retinal disease remains uncertain. In a model of subretinal fibrosis, Seyed-Razavi et al. (2024) observed extension of Müller glial processes into lesioned areas together with increased expression of profibrotic factors, including α-SMA, fibronectin, and connective tissue growth factor. These observations are consistent with the involvement of reactive Müller glia in the fibrotic microenvironment but do not independently establish that Müller glia are the cellular source of all profibrotic markers or that they undergo complete GMT *in vivo*. Similarly, Wu et al. (2020) and Palko et al. (2023) reported co-expression of glial markers, including GFAP and GS, with mesenchymal-associated markers such as SM22 and SNAIL in fibrovascular tissues from patients with diabetic retinopathy, particularly PDR. Such co-expression is compatible with a partial or hybrid GMT-like state in which Müller glia retain aspects of their glial identity while acquiring selected mesenchymal features. Several transcription factors may participate in this process, including Snail (SNAI1), Slug (SNAI2), and forkhead box O1 (FOXO1) (Chen et al., 2019; Odarenko et al., 2024). Snail and Slug are members of the SNAIL family of transcriptional repressors that promote mesenchymal transition by repressing cell-identity and adhesion-associated genes and facilitating cytoskeletal remodeling and migration (Stemmler et al., 2019). In Müller glia, activation of the TGF-β–SNAIL axis has been associated with increased ECM synthesis, contractility, and cell migration (Kanda et al., 2019). FOXO1 is a stress- and metabolism-responsive transcription factor whose activity is regulated by PI3K–AKT signaling and oxidative stress (Webb and Brunet, 2014). However, its effects on mesenchymal and fibrotic programs in Müller glia require further validation. Collectively, these findings support substantial Müller glial plasticity and the presence of partial or hybrid GMT-like phenotypes. However, marker co-expression alone cannot determine whether these states are transitional, stable, reversible, or functionally profibrotic. Longitudinal lineage-tracing and functional studies are therefore required to establish their cellular origin, temporal progression, and causal contribution to retinal fibrosis.

The functional consequences of Müller cell GMT may vary with disease context, the nature and duration of profibrotic stimulation, and the persistence of GMT-like states. By analogy with myofibroblast responses during tissue injury, an early and limited GMT-like response could hypothetically contribute to wound containment, provisional ECM formation, or structural remodeling (Wynn and Ramalingam, 2012). In contrast, persistent TGF-β signaling may promote contractile and ECM-synthesizing phenotypes in Müller glia, whereas inflammatory signals and mechanical feedback from the remodeled ECM may further sustain profibrotic cellular states (Kanda et al., 2019; Pakshir et al., 2018). Direct evidence demonstrating an adaptive early phase or reversibility of Müller cell GMT *in vivo* nevertheless remains limited, and this proposed adaptive-to-maladaptive progression should be regarded as a working model. GMT should also be considered within the multicellular context of retinal fibrosis, which, depending on the disease and anatomical compartment, may involve EMT of RPE cells, endothelial-to-mesenchymal transition (EndMT), and MMT (Shu et al., 2020; Little et al., 2020). EndMT refers to the acquisition of mesenchymal-like properties by endothelial cells, whereas MMT describes the acquisition of myofibroblast-like features by macrophages. These transition programs may be regulated by overlapping profibrotic signals, including TGF-β, inflammatory mediators, oxidative stress, and mechanical feedback (Kanda et al., 2019; Shu et al., 2020; Little et al., 2020). Current evidence does not establish GMT as an independent or predominant driver of retinal fibrosis; rather, it may represent one component of a broader multicellular fibrotic network. Nevertheless, the radial distribution and extensive cellular interactions of Müller glia, together with evidence from iERM and PDR tissues, suggest that GMT-like remodeling may be particularly relevant to epiretinal fibrocellular or fibrovascular membrane formation (Pakshir et al., 2018). Whether selective targeting of GMT offers advantages over targeting other cellular transitions or their shared upstream pathways remains to be established and will require Müller cell-specific interventions and *in vivo* validation.

## 4 Retinal fibrosis-related signaling pathways

The acquisition of mesenchymal-like and fibrogenic features by Müller glia is thought to involve a complex and interconnected regulatory network. Profibrotic, inflammatory, oxidative, metabolic, and mechanical cues within the diseased retinal microenvironment may influence the emergence and persistence of GMT-like states. However, the mechanisms governing the progression from reactive gliosis to partial or persistent Müller cell GMT remain incompletely understood. Current evidence particularly implicates TGF-β, Notch, inflammatory, and oxidative stress-related signaling, together with additional biomechanical and metabolic mechanisms. The following section discusses these pathways and critically evaluates the evidence linking them to Müller cell GMT and retinal fibrotic remodeling.

### 4.1 TGF-β signaling pathway

Transforming growth factor-β (TGF-β) is a multifunctional cytokine involved in development, immune regulation, tissue repair, and fibrosis. Mammals express three TGF-β isoforms—TGF-β1, TGF-β2, and TGF-β3—which are synthesized as latent complexes and can be activated in response to tissue injury and changes in the extracellular microenvironment (Hata and Chen, 2016; Derynck and Budi, 2019). In the canonical pathway, ligand-bound TGF-β receptor type II recruits and phosphorylates TGF-β receptor type I, also known as activin receptor-like kinase 5 (ALK5), resulting in the phosphorylation of SMAD2 and SMAD3. Activated SMAD2/3 forms a complex with SMAD4 and translocates to the nucleus, where it regulates target-gene transcription in cooperation with other transcriptional regulators (Hata and Chen, 2016; Derynck and Budi, 2019). Depending on the cellular context, TGF-β may also engage non-canonical pathways, including MAPK, PI3K–AKT and NF-κB-associated signaling, thereby linking profibrotic signaling with inflammatory and cellular stress responses (Derynck and Budi, 2019). In Müller glia, both TGF-β1 and TGF-β2 have been shown to induce GMT-associated molecular changes, making TGF-β one of the best-supported candidate regulators of Müller cell GMT (da Silva et al., 2023; Wu et al., 2020).

The TGF-β–SNAIL axis is particularly relevant to the acquisition of GMT-like features. In MIO-M1 cells, TGF-β1 and TGF-β2 increased the expression of ACTA2/α-SMA, TAGLN/SM22, COL1A1, and fibronectin, while TGF-β1 also increased SNAIL expression, cellular migration, proliferation, and collagen-gel contraction and reduced the Müller glial marker glutamine synthetase (Kanda et al., 2019). siRNA-mediated knockdown of SNAI1 attenuated these molecular and functional changes, supporting a regulatory role for SNAIL downstream of TGF-β *in vitro* (Kanda et al., 2019). In addition, Müller-associated cells expressing TGF-β receptors, phosphorylated SMAD2, SNAIL, and mesenchymal-associated markers have been detected in surgically excised epiretinal tissues from patients with iERM or PDR (Kanda et al., 2019; Wu et al., 2020). These findings support the clinical relevance of the pathway but do not establish Müller cell lineage or complete GMT *in vivo*. Wu et al. further showed that TGF-β1 and TGF-β2 increased TGFB1 and TGFB2 expression in cultured Müller cells through non-canonical signaling involving NF-κB, p38 MAPK, and GSK-3, suggesting a potential autocrine feed-forward mechanism that could reinforce TGF-β signaling (Wu et al., 2020). Notably, SMAD2 knockdown did not abolish this autoinduction, whereas TGF-β-induced VEGF-A expression involved SMAD2, p38 MAPK, and PI3K–AKT signaling, indicating that TGF-β autoinduction and VEGF-A production are mediated by partly distinct signaling branches (Wu et al., 2020). The latter observation provides a possible molecular link between profibrotic and angiogenic responses, although its contribution to the angiofibrotic switch *in vivo* remains uncertain. Pharmacological inhibition of ALK5 with galunisertib reduced SMAD3 phosphorylation and attenuated TGF-β1-induced GMT-like molecular and contractile changes in MIO-M1 cells (da Silva et al., 2023). Collectively, these studies identify TGF-β signaling as a major candidate regulator of Müller cell GMT. Nevertheless, most mechanistic and intervention evidence is derived from immortalized cell lines, whereas patient-tissue evidence is primarily based on marker co-localization. Müller cell-specific *in vivo* studies are therefore required to determine whether the TGF-β–SNAIL axis is necessary or sufficient for retinal fibrosis and whether its selective inhibition can prevent, attenuate, or reverse established fibrotic remodeling.

### 4.2 Notch signaling pathway

The Notch signaling pathway is an evolutionarily conserved intercellular communication mechanism. Binding of Jagged or Delta-like ligands to NOTCH1–4 receptors induces sequential proteolytic cleavage by ADAM-family metalloproteases and the γ-secretase complex, releasing the Notch intracellular domain (NICD). NICD translocates to the nucleus and associates with RBPJ/CSL and mastermind-like co-activators to regulate downstream genes, including members of the HES and HEY families, thereby modulating cell differentiation, proliferation, and fate determination (Hu and Phan, 2016; Samer et al., 2024; Shao et al., 2015; Bray, 2006). In fibrotic conditions, dysregulated Notch signaling may interact with TGF-β signaling and promote EMT- or EndMT-associated phenotypic remodeling and ECM accumulation, although its effects are dependent on cell type and disease context (Shao et al., 2015; Shafiee et al., 2017; Chen et al., 2014). In RLE-6TN alveolar epithelial cells, TGF-β1 increased Notch1, NICD, HES1, and TWIST1 expression and induced EMT-associated changes, including reduced E-cadherin and ZO-1 and increased vimentin and N-cadherin expression (Tang et al., 2021). Notch1 silencing attenuated these changes, supporting the involvement of the Notch1–TWIST1 axis in TGF-β1-induced pulmonary epithelial remodeling (Tang et al., 2021). However, because this evidence was obtained from a pulmonary epithelial model, it provides mechanistic background rather than direct evidence of Müller cell GMT.

In Müller glial cells, Notch signaling regulates proliferation, survival, quiescence, and injury responses. In zebrafish retinal injury models, the Notch3–DeltaB axis helps maintain Müller glial quiescence, whereas other Notch receptors may support injury-induced proliferation, indicating receptor- and stage-dependent effects (Campbell et al., 2021; Campbell et al., 2022). Evidence directly linking Notch signaling to Müller cell GMT remains limited. Fan et al. (2020) showed that Notch ligands activated downstream Notch effectors and increased endogenous TGF-β1, TGF-β receptors, SMAD3 phosphorylation, and ECM-associated protein expression in MIO-M1 cells. Combined stimulation with Notch ligands and TGF-β1 produced additive increases in ECM-associated proteins, which were attenuated by RO4929097, a γ-secretase inhibitor (Fan et al., 2020). In a mouse model of sodium iodate-induced retinal injury, intravitreal RO4929097 reduced Müller glial reactivity and ECM-protein overexpression, with no overt short-term retinal toxicity detected during the 17-day observation period (Fan et al., 2020). However, these findings do not independently demonstrate Müller cell GMT or myofibroblast formation *in vivo*. Therefore, current evidence supports Notch–TGF-β crosstalk in Müller cell-associated ECM remodeling but does not establish Notch signaling as an independent or Müller cell-specific driver of GMT. Further cell-specific and lineage-tracing studies are required to clarify its precise role in retinal fibrosis.

### 4.3 Inflammatory factors

Inflammation is an important component of retinal fibrotic remodeling. Persistent metabolic and oxidative stresses, including hyperglycemia, reactive oxygen species (ROS), and advanced glycation end products (AGEs) in diabetic retinopathy, can activate Müller glia, RPE cells, vascular endothelial cells, and resident or infiltrating immune cells (Eastlake et al., 2016; Tang et al., 2023; Sisto et al., 2021). The resulting cytokine- and chemokine-rich microenvironment, involving mediators such as IL-6, IL-8, MCP-1, and IL-1β, may modulate EMT-, GMT-, and EndMT-associated signaling, promote cellular migration and contractility, and alter ECM remodeling, thereby sustaining bidirectional crosstalk between inflammation and fibrosis (Jo et al., 2011; Jing et al., 2019).

Current evidence suggests that specific inflammatory mediators can modulate Müller glial reactivity and GMT-like remodeling. Song et al. (2023) reported elevated IL-4 levels in patients with idiopathic epiretinal membrane and found that IL-4 promoted the proliferation and migration of cultured primary rat Müller glia while modulating the expression of GMT-associated molecules, including COL1A1, fibronectin, α-SMA, and GFAP. These effects appeared to be independent of TGF-β1, suggesting that inflammatory mediators may influence Müller glial phenotypes through mechanisms beyond canonical TGF-β signaling. However, the clinical findings are primarily associative, and the mechanistic evidence is largely derived from cultured cells. The contribution of IL-4 to Müller cell GMT and fibrosis *in vivo* therefore requires further validation. In epithelial models, IL-6 and IL-8 can regulate transcription factors such as SNAIL and TWIST through JAK/STAT3 and NF-κB signaling, thereby promoting EMT-associated migration and fibrotic phenotypes (Eastlake et al., 2016; Wang et al., 2002). However, these findings cannot be directly extrapolated to Müller glia. Li et al. provided more direct retina-related evidence linking JAK2/STAT3 signaling to profibrotic responses in Müller glia. Under diabetic conditions, apelin-mediated JAK2/STAT3 activation was associated with increased expression of α-SMA, type I collagen, and other fibrosis-related molecules in Müller glia, whereas apelin knockdown or STAT3 inhibition attenuated these changes (Li et al., 2023). Nevertheless, the role of STAT3 in Müller glia is strongly context-dependent. In a mammalian retinal injury model, JAK/STAT3 activation was associated with Müller glial reactivity and increased GFAP expression, whereas pharmacological STAT3 inhibition reduced GFAP expression in retinal explants (Tassoni et al., 2015). By contrast, STAT3 is required for the full proliferative and regenerative response of Müller glia following retinal injury in zebrafish (Nelson et al., 2012). STAT3 activation should therefore not be classified as exclusively inflammatory, reparative, or profibrotic; its functional consequences likely depend on the upstream stimulus, disease stage, species, and local microenvironment. Moreover, the study by Li et al. primarily demonstrated changes in reactive and fibrosis-associated markers and did not comprehensively establish the loss of glial markers, acquisition of multiple mesenchymal features, associated functional changes, or Müller cell lineage conversion *in vivo* (Li et al., 2023). Current evidence therefore supports a potential role for JAK2/STAT3 signaling in modulating the profibrotic activity of Müller glia but does not establish that STAT3 activation is sufficient to initiate Müller cell GMT. NF-κB may similarly connect inflammatory responses with profibrotic remodeling. In RPE and other epithelial cells, NF-κB can promote mesenchymal-like remodeling by regulating transcription factors such as SNAIL and ZEB1 (Yoo et al., 2021; Wu et al., 2020). In cultured human Müller cells, NF-κB participates in TGF-β autoinduction associated with GMT-related molecular changes, suggesting crosstalk between inflammatory transcriptional signaling and profibrotic reprogramming (Wu et al., 2020). However, these findings are derived primarily from *in vitro* systems and do not demonstrate that NF-κB activation alone is sufficient to induce Müller cell GMT or retinal fibrosis *in vivo*. Collectively, inflammatory mediators may contribute to retinal fibrosis by establishing a profibrotic microenvironment and modulating reactive and GMT-like states in Müller glia. Nevertheless, inflammatory pathway activation, reactive gliosis, and GMT should not be regarded as interchangeable processes.

### 4.4 Oxidative stress

Oxidative stress is an important component of the pathological microenvironment associated with Müller glial dysfunction. In cultured rat Müller cells, high glucose increases ROS production, transiently reduces Nrf2 expression and its downstream antioxidant responses, and subsequently activates NF-κB-associated inflammatory signaling (Albert-Garay et al., 2022). High glucose can also disturb glutathione homeostasis, antioxidant-enzyme activity, and mitochondrial function, although the magnitude and timing of these responses vary among experimental models (Carpi-Santos et al., 2022; Albert-Garay et al., 2022). More recently, the AQP4–TRPV4 axis has been implicated in Müller cell ferroptosis under diabetic conditions. AQP4 and TRPV4 were upregulated in high-glucose-treated Müller cells and diabetic rat retinas, whereas inhibition of AQP4 or TRPV4 reduced oxidative stress, lipid peroxidation-associated changes, ferroptosis, and apoptosis (Chen et al., 2024a). These findings establish redox dysregulation and ferroptotic susceptibility in Müller cells but do not independently demonstrate GMT.

Oxidative stress may nevertheless influence Müller cell GMT indirectly by sustaining inflammatory signaling and modifying cellular responsiveness to profibrotic mediators such as TGF-β. Increased GFAP and vimentin expression and Müller cell hypertrophy under retinal injury conditions reflect reactive gliosis rather than mesenchymal transition. Although RNAi-mediated suppression of vimentin or GFAP attenuated Müller glial hypertrophy in a model of progressive retinal degeneration, this finding does not establish a direct link between oxidative stress and GMT (Hippert et al., 2021). Similarly, BMSC-derived exosomal miR-486–3p reduced oxidative stress, inflammation, and apoptosis in high-glucose-treated Müller cells through repression of TLR4/NF-κB signaling (Li et al., 2021), while 670 nm photobiomodulation reduced ROS production, preserved mitochondrial integrity, and attenuated NF-κB activation and ICAM-1 expression in high-glucose-treated rMC-1 cells (Nonarath et al., 2021). However, neither intervention was evaluated using a comprehensive set of GMT-related molecular and functional endpoints. Collectively, current evidence supports oxidative stress as a potential permissive or amplifying factor that links metabolic injury, inflammation, and Müller glial reactivity, but direct evidence that oxidative stress initiates or stabilizes Müller cell GMT remains limited. Future studies should determine whether redox manipulation alters glial-marker loss, mesenchymal-marker acquisition, migration, contractility, and ECM remodeling in Müller cells *in vivo*.

### 4.5 Other mechanisms

In addition to TGF-β, Notch, inflammatory, and oxidative stress pathways, Müller cell GMT may be influenced by complementary biomechanical and metabolic mechanisms. Mechanical stress and ECM stiffening can activate integrin–FAK signaling and the Hippo–YAP/TAZ axis, promoting the transcription of profibrotic genes such as *ACTA2*, *CTGF*, and collagens and potentially favoring the persistence of contractile, mesenchymal-like phenotypes (Liu et al., 2015; Panciera et al., 2017). Aberrant Wnt/β-catenin signaling may further reinforce mesenchymal gene expression and interact with Hippo signaling during fibrotic remodeling (Zhao et al., 2024; Jiang et al., 2025; Gorsuch et al., 2017). Metabolic reprogramming toward glycolysis, mitochondrial dysfunction, ER stress-associated unfolded protein responses, and matricellular proteins such as osteopontin and periostin may also modulate cytoskeletal remodeling, ECM homeostasis, and profibrotic signaling (Yin et al., 2025; Zhang et al., 2020; Too et al., 2017). Extracellular proteolysis may also shape the retinal fibrotic microenvironment. In a two-stage laser-induced mouse model, Granzyme B deficiency reduced subretinal fibrosis in older mice, whereas exogenous Granzyme B promoted partial EMT-related changes in cultured ARPE-19 cells (Yoo et al., 2025). GFAP-positive macroglial activation was also observed above the fibrotic lesions, consistent with reactive gliosis. Although these findings suggest an association with extracellular matrix remodeling and retinal fibrosis, the direct roles of several of these mechanisms in Müller cell GMT remain incompletely established. Collectively, these mechanisms may interact with established profibrotic pathways and represent potential targets for further investigation in retinal fibrosis.

## 5 Müller cell GMT in retinal diseases and therapeutic implications

Müller cell–driven GMT is increasingly recognized as a potential convergent mechanism underlying various forms of retinal fibrosis. Although these conditions differ in their clinical manifestations—ranging from epiretinal membrane formation to subretinal fibrosis following choroidal neovascularization—the core pathological features, including excessive ECM deposition, contractile membrane formation, and prolonged tissue remodeling, are frequently associated with injury-induced phenotypic reprogramming of Müller glial cells.

### 5.1 Proliferative diabetic retinopathy

Proliferative diabetic retinopathy (PDR) is an advanced form of diabetic retinopathy characterized primarily by retinal ischemia-associated neovascularization and the formation of preretinal fibrovascular membranes (Lim et al., 2025). The fragile neovessels are prone to vitreous hemorrhage, whereas persistent remodeling and contraction of fibrovascular membranes can exert traction on the retina, potentially leading to tractional retinal detachment and irreversible visual impairment. PDR-associated fibrovascular membranes comprise multiple cell types and ECM components, and their formation involves complex interactions among angiogenic, inflammatory, and fibrotic processes (Lim et al., 2025). Within this multicellular pathological context, Müller glia are considered one of the potential cellular contributors.

The association between Müller glia and fibrotic remodeling in PDR has been established progressively over several decades. Nork et al. (1987) identified Müller glia in preretinal membranes from patients with PDR using carbonic anhydrase histochemistry and GFAP immunolabeling. They also observed displacement of Müller cell nuclei, intraretinal bridging structures, and membrane-like formations, suggesting that Müller glia may actively participate in proliferative tissue remodeling in PDR. Guidry et al. (2009) subsequently reported that cultured Müller glia and Müller glia within PDR fibrovascular membranes exhibited reduced GS and GFAP expression, increased α-SMA expression, and a fibroblast-like morphology, supporting their potential to acquire a fibrocontractile phenotype. Zhou et al. (2017) further demonstrated that high-glucose exposure enhanced Müller cell migration and increased the expression of mesenchymal-associated molecules, including SNAIL, N-cadherin, vimentin, and α-SMA. SNAIL also regulated the expression of CTGF and fibronectin, thereby linking the diabetic microenvironment to mesenchymal-like remodeling of Müller glia. Within the conceptual framework of GMT, Wu et al. (2020) subsequently demonstrated changes involving Müller glial markers, SNAIL, SM22, and TGF-β signaling in PDR fibrovascular tissues and cultured Müller glia. They further proposed that TGF-β autoinduction may promote both GMT-like changes and VEGF-A production. Collectively, these studies provide a progressive body of evidence encompassing cellular localization, fibrocontractile phenotypes, mesenchymal-marker expression, and TGF-β-related mechanisms. This evidence supports an association between Müller cell GMT-like remodeling and fibrovascular proliferation and fibrotic remodeling in PDR; however, it does not yet establish Müller cell GMT as an independent causal driver of PDR-associated fibrosis.

TGF-β is considered one of the major candidate pathways regulating GMT-like changes in Müller glia. Histopathological studies of PDR FVMs have detected activation of TGF-β-associated signaling together with altered expression of Müller glial and mesenchymal-associated markers, including GS, GFAP, and α-SMA (da Silva et al., 2023; Wu et al., 2020). Experimental studies further indicate that TGF-β-induced GMT-like remodeling can increase Müller glial contractility and alter ECM synthesis or remodeling, potentially contributing to the formation and contractile properties of FVMs (Wu et al., 2020; Chang et al., 2018). However, these changes have not been shown to be sufficient to cause tractional retinal detachment *in vivo*. Retinal traction depends on the cellular and ECM composition, spatial organization, retinal adhesion, and biomechanical properties of FVMs (Iyer et al., 2019; Scheri et al., 2023). TGF-β stimulation of Müller glia has also been associated with GMT-related changes and increased VEGF-A production, suggesting potential crosstalk between fibrotic and angiogenic signaling (Wu et al., 2020; Rezzola et al., 2021). Together with their inflammatory and ECM-remodeling responses, these findings suggest that Müller glia may participate in the angiofibrotic switch, which describes a disease-associated shift from angiogenic activity toward fibrotic remodeling in PDR and neovascular AMD (Yoo et al., 2025; Roberts et al., 2019). However, Müller cell GMT has not been established as a causal driver of this switch, which likely involves multiple retinal, vascular, immune, and stromal populations. Evidence that fibrosis may develop or progress despite anti-VEGF treatment supports further investigation of strategies addressing both angiogenic and fibrotic remodeling (Yoo et al., 2025; Roberts et al., 2019).

### 5.2 Idiopathic epiretinal membrane

Idiopathic epiretinal membrane (iERM) is a fibrocellular proliferative disorder that develops on the retinal surface of the macular region. Multiple cell populations have been identified in iERM tissues, including microglia, Müller glia, astrocytes, hyalocytes, retinal pigment epithelial cells, and myofibroblasts (da Silva et al., 2022). Within this multicellular context, GMT-like remodeling of Müller glia has been proposed as one potential mechanism associated with iERM development. Bu et al. (2015) detected Müller cell-associated markers, α-SMA, and collagens in human iERM tissues and showed that TGF-β1 induced α-SMA-positive stress-fiber formation and phenotypic changes in cultured Müller glia, providing early evidence that these cells can acquire myofibroblast-like features. iERM was subsequently linked more explicitly to Müller cell GMT by Kanda et al. (2019). They detected Müller glial markers together with GMT-associated molecules in iERM tissues and showed that TGF-β1 induced SNAIL-dependent GMT-like molecular and functional changes in MIO-M1 cells. Krishna Chandran et al. (2021) further linked the intraocular microenvironment of patients with iERM to Müller cell GMT. Vitreous samples from patients with iERM promoted MIO-M1 cell proliferation and migration and induced varying degrees of glial-marker loss and mesenchymal- or myofibroblast-associated marker expression. The responses differed among vitreous samples (Krishna Chandran et al., 2021): some induced both downregulation of glial markers and upregulation of mesenchymal markers, whereas others induced glial-marker downregulation without a corresponding increase in mesenchymal markers (Krishna Chandran et al., 2021). These findings suggest interindividual variability and potentially heterogeneous states of Müller cell remodeling in iERM. Song et al. (2023) subsequently extended the proposed regulatory framework to inflammatory signaling. They found elevated aqueous humor IL-4 levels in patients with iERM, which were associated with postoperative changes in macular thickness and volume following cataract surgery (Song et al., 2023). *In vitro*, IL-4 promoted the proliferation and migration of primary rat Müller glia and modulated the expression of GMT-associated molecules, including COL1A1, fibronectin, α-SMA, and GFAP, potentially through a TGF-β1-independent mechanism (Song et al., 2023). These findings suggest that inflammatory mediators, in addition to the canonical TGF-β–SNAIL axis, may contribute to iERM-associated Müller cell remodeling. Collectively, current evidence indicates that Müller glia can acquire GMT-like molecular and functional features under iERM-associated conditions and that these responses may be regulated by IL-4, TGF-β1, and other vitreous-derived factors. However, iERM is a multicellular disease, and most mechanistic evidence is derived from immortalized Müller cell lines or primary cells maintained *in vitro*. The causal role and quantitative contribution of Müller cell GMT to iERM therefore remain uncertain. Müller cell-specific lineage-tracing and functional intervention studies will be required to determine whether GMT causally contributes to iERM formation and whether its selective inhibition has therapeutic value.

### 5.3 Other retinal diseases

Beyond the well-characterized conditions, accumulating but fragmented evidence suggests that GMT-like remodeling may also occur in several less extensively studied retinal disorders. Proliferative vitreoretinopathy (PVR) represents a prominent example: following rhegmatogenous retinal detachment, Müller glial cells may migrate through retinal breaks into the vitreous cavity and, under persistent stress, acquire an α-SMA–positive myofibroblast-like phenotype (Feist et al., 2014; Harju et al., 2025). In neovascular age-related macular degeneration (nAMD), Müller cells have been proposed as a potential source of myofibroblasts in subretinal fibrosis, although direct evidence for Müller cell GMT remains limited; excessive ECM deposition within fibrotic scars disrupts retinal architecture and contributes to irreversible central vision loss (Tenbrock et al., 2022). In addition, fibrotic lamellar structures associated with Müller glial cells have been observed in high myopia–related macular pathology (Chen et al., 2024b). Emerging evidence from experimental myopia identifies a Fos-positive Müller glial subpopulation with GMT-associated features, implicating the IGF2–ERK–Fos axis in Müller cell GMT and myopia-associated retinal remodeling (Feng et al., 2026). Although the available evidence remains limited, persistent Müller cell GMT-like changes may contribute to fibrotic remodeling in retinal disorders associated with chronic or recurrent injury.

### 5.4 Therapeutic implications and delivery considerations

Pharmacological targeting of Müller cell GMT remains at an early experimental stage. The most direct evidence involves inhibition of TGF-β signaling. Galunisertib, an ALK5 inhibitor, has been shown to attenuate TGF-β1-induced GMT-like changes in cultured Müller cells (da Silva et al., 2023). Other compounds have also been evaluated in diabetic or profibrotic culture conditions. Ginsenoside Rg1 attenuated high glucose-induced mesenchymal activation through the miR-2113/RP11-982M15.8/ZEB1 pathway, reducing mesenchymal-marker and MMP-2/MMP-9 expression while restoring TIMP-2 expression (Xue et al., 2018). Erythropoietin (EPO) similarly attenuated the acquisition of fibrotic features and modulated the migratory behavior of Müller glia exposed to TGF-β and high glucose (Luo et al., 2016). Beyond TGF-β-associated interventions, the γ-secretase inhibitor RO4929097 reduced Notch/TGF-β-associated ECM protein expression in cultured Müller cells, while intravitreal administration attenuated Müller gliosis and ECM accumulation in a sodium iodate-induced mouse model (Fan et al., 2020). Experimental YAP inhibition with verteporfin also suppressed TGF-β- and matrix stiffness-induced myofibroblast-like changes in cultured human Müller cells and reduced retinal fibrotic changes in diabetic rats (Zhang and Kong, 2020). However, verteporfin is not a selective YAP inhibitor, and the *in vivo* effects of RO4929097 and verteporfin cannot be attributed specifically to inhibition of Müller cell GMT. Intravitreal injection currently provides the most direct route for delivering experimental agents to the neural retina and has been used for RO4929097 (Fan et al., 2020). Sustained-release implants, hydrogels, nanoparticles, and periocular or suprachoroidal administration may potentially prolong local drug exposure or improve delivery to posterior ocular tissues, whereas viral or nonviral gene delivery could theoretically enable longer-term and more cell-selective regulation (Varela-Fernández et al., 2020; Habot-Wilner et al., 2019). However, these approaches have not been validated specifically for targeting Müller cell GMT in retinal fibrosis, and conventional eye drops generally provide insufficient posterior-segment exposure (Rodrigues et al., 2018). Overall, much of the pharmacological evidence is derived from cultured Müller cells and may not reproduce their polarity, ECM interactions, or multicellular signaling within the intact retina. It therefore remains unknown whether galunisertib, Rg1, EPO, RO4929097, verteporfin, or other interventions can prevent or reverse retinal fibrosis by selectively suppressing Müller cell GMT *in vivo*. Future studies should evaluate ocular pharmacokinetics, Müller cell specificity, therapeutic timing, retinal safety, and cell-specific antifibrotic efficacy.

## 6 Conclusion

Müller cell GMT has emerged as an important cellular reprogramming process increasingly implicated in retinal fibrotic diseases. Growing evidence suggests that GMT is associated with the morphological, phenotypic, and functional changes observed during retinal fibrosis and may contribute to fibrotic remodeling. However, current studies have not established a definitive causal relationship between Müller glial GMT and retinal fibrosis. Most available evidence is derived from immunohistochemical analyses, *in vitro* culture systems, or immortalized Müller glial cell lines, which primarily demonstrate the acquisition of mesenchymal characteristics rather than proving that GMT is a primary driver of fibrotic tissue formation *in vivo*. Furthermore, retinal fibrosis is a complex multicellular process involving RPE cells undergoing EMT, endothelial cells, infiltrating macrophages, fibroblasts, and other stromal populations. Consequently, the relative contribution of Müller glial GMT to retinal fibrotic remodeling remains to be fully elucidated.

Several significant knowledge gaps remain. The mechanisms governing the progression from reactive gliosis to partial or persistent GMT-like states remain poorly understood, and it is unknown whether GMT occurs broadly across reactive Müller glia or is preferentially restricted to specific reactive subpopulations, including reparative or profibrotic states. No Müller glial subpopulation with a preferential propensity for GMT has yet been identified. In addition, the molecular regulatory architecture of GMT remains incompletely understood, particularly the roles of TGF-β and Notch signaling, their crosstalk with inflammatory, metabolic, oxidative, and mechanical signals, and the contribution of epigenetic mechanisms such as chromatin remodeling, DNA methylation, and histone modifications. Current experimental models also have inherent limitations: murine models exhibit species-specific differences from human Müller glial biology, whereas primary human Müller glia are difficult to isolate and maintain, and immortalized cell lines such as MIO-M1 may not reproduce cell polarity, ECM interactions, and multicellular crosstalk *in vivo*. Future studies integrating single-cell and spatial transcriptomics, Müller cell-specific lineage tracing, cell type-specific genetic manipulation, and physiologically relevant *in vivo* models will therefore be essential to identify reactive Müller glial states that may precede GMT, establish its causal contribution to retinal fibrosis, and determine whether selective GMT inhibition can prevent, attenuate, or reverse fibrotic remodeling. Despite these challenges, the GMT concept has expanded our understanding of Müller glial plasticity and may provide new perspectives for identifying antifibrotic therapeutic targets in retinal disease.
